# Die Einstellung von Patienten in geriatrischer Rehabilitation zu Pflegeheimen

**DOI:** 10.1007/s00391-020-01773-8

**Published:** 2020-08-17

**Authors:** Natalie Schwind, Georg Jahn

**Affiliations:** Professur Angewandte Gerontopsychologie und Kognition, Institut für Psychologie, Technische Universität Chemnitz, Wilhelm-Raabe-Str. 43, 09120 Chemnitz, Deutschland

**Keywords:** Heimeinzug, Entscheidung, Erfahrung, Informationsstand, Kontrollerleben, Relocation, Decision, Experience, Level of information, Feeling in control

## Abstract

**Hintergrund:**

Mit Erreichen des höheren Lebensalters nehmen unterstützende Wohnformen eine immer wichtigere Rolle ein. Eingeschränkte Selbstständigkeit kann dazu führen, dass ein Umzug in ein Pflegeheim unabdingbar ist. Die Einstellung zu Pflegeheimen ist von großer Bedeutung sowohl für die Vorbereitung auf diesen Fall als auch für die psychische Reaktion auf sein Eintreten, wurde allerdings bisher kaum näher beforscht.

**Ziel der Arbeit (Fragestellung):**

Das Ziel dieser Untersuchung ist herauszufinden, wie Pflegeheime von älteren Menschen je nach ihrem subjektiven Kontrollerleben, ihrem Informationsstand und ihren Erfahrungen bewertet werden.

**Material und Methoden:**

Zu Einstellungen gegenüber Pflegeheimen wurden insgesamt 150 geriatrische Rehabilitationspatienten mit einer ersten Fragebogenversion (*n* = 64) oder einer revidierten Fassung (*n* = 86) befragt.

**Ergebnisse:**

Mittels Polaritätsprofil konnte eine eher positive Einstellung zu Pflegeheimen beobachtet werden, jedoch wurden bei freiem Antwortformat überwiegend negative Assoziationen geäußert. Der Großteil der Probanden hat eine ängstliche Haltung zu einem Umzug in ein Pflegeheim. Stärkeres Kontrollerleben, ein besserer Informationsstand und positive Erfahrungen mit Pflegeheimen stehen mit einer positiven Haltung zum Umzug in ein Pflegeheim im Zusammenhang.

**Diskussion:**

Mit Blick auf geäußerte Ängste, einen verbreitet geringen Informationsstand und die starke Abwehrhaltung einiger Befragter gilt es, die Betroffenen proaktiv zu informieren und stärker in die Entscheidungen für eine Wohnoption einzubinden. Neueste Entwicklungen zu aussagekräftigerer Qualitätsdarstellung der Pflegeheime können dabei unterstützen.

## Abstract (en)

**Background:**

For the group of individuals reaching higher age, living arrangements providing care increase in importance. Increasing dependence can lead to a relocation to a nursing home. The attitude towards nursing homes is important in preparing for this event and the psychological response to it but has hardly been studied so far.

**Objective:**

The aim of this study was to determine what older people think about and how they evaluate nursing homes depending on their subjective feeling of control, their experiences and on how informed they are.

**Material and methods:**

A total of 150 geriatric rehabilitation patients were interviewed on their attitudes toward nursing homes with a first version of a questionnaire (*n* = 64) or a revised version (*n* = 86).

**Results:**

A polarity profile showed a more positive attitude toward nursing homes; however, the participants gave mainly negative statements in a free response format. The majority of the participants had an anxious attitude towards a relocation to a residential home. A stronger feeling of control, a higher level of information, and positive experiences with nursing homes are related to a positive attitude towards the relocation to a nursing home.

**Conclusion:**

Given the named fears, a common low level of information, and a great defensiveness of some participants, it is important to proactively inform older people and to involve them more closely in the decision process for suitable living arrangements. Recent developments towards more reliable quality assessment of nursing homes can be helpful.

Im Zuge des demografischen Wandels wächst auch in Deutschland die Zahl der Menschen, die ihren letzten Lebensabschnitt im Pflegeheim verbringen [14]. Im Vorfeld eines Umzugs in diese institutionalisierte Wohnform, bei der Planung eines Umzugs und für die Integration in die neuen Wohnverhältnisse ist die Einstellung gegenüber Pflegeheimen von Bedeutung. Für Patienten in geriatrischer Rehabilitation kann ein Heimeinzug unmittelbar oder in absehbarer Zeit anstehen; sie können auch als Mitverantwortliche (z. B. als Partner) in die Planung von Pflegearrangements für andere Personen einbezogen sein. Die Einstellungen in dieser Gruppe sind daher von besonderer Relevanz, um den Bedarf nach Interventionen abzuschätzen.

## Hintergrund

Stereotype Ansichten, dass Heimbewohner „nur rumsitzen und nichts tun“ oder „in den Gemeinschaftsraum gefahren werden und für den restlichen Tag dort verweilen“, spiegeln Ängste und Erwartungen vieler Älterer wider [10]. Meist fällt der Vergleich zwischen Pflegeheim und dem Leben zu Hause ungünstig für die institutionelle Wohnform aus [17]. Eine Befragung im Rahmen des DAK(Deutsche Angestellten Krankenkasse)-Pflegereports (2018) zeigt, dass 62 % der Befragten am liebsten zu Hause durch den Partner oder andere Angehörige bei Pflegebedarf bereut werden möchten. Nur 5 % der Befragten möchten im Pflegeheim leben, wenn sie auf Pflege angewiesen sind [5].

Die heute Älteren haben andere Ansprüche an die Versorgung [12]. Ihnen sind vermehrt ein selbstbestimmtes Leben, die freie Wahl des Wohnumfelds und ein Mitspracherecht in der Pflege wichtig [12]. Zu Hause zu leben, ob nun allein, mit dem Partner oder in einer Wohngemeinschaft ist dabei die Idealvorstellung [6, 21]. Im privaten Haushalt leben 97 % der 60-Jährigen und 90 % der über 80-Jährigen [14]. Stationär versorgt werden 24 % der Pflegebedürftigen [24], bei steigender Pflegebedürftigkeit [26]. Die Zahl der Heime stieg seit 2015 um 6,5 % an [24].

Bei der Entscheidung für oder gegen den Umzug in eine stationäre Einrichtung spielen mehrere Faktoren eine Rolle. Die finanzielle Situation und das aktuelle Wohnumfeld bestimmen die Möglichkeiten der Versorgung zu Hause. Wenn der Pflegebedarf älterer Personen steigt, sie sich im Privathaushalt nicht mehr sicher fühlen, die Mühe der Selbstversorgung verringern wollen oder Ärzte und Angehörige zu einem Umzug raten [21], dann wird der Heimeinzug wahrscheinlich. In einer bevölkerungsrepräsentativen Umfrage zeigten sich bei der Einschätzung von möglichen Pflegearrangements für Personen mit Demenz eine geringere Tendenz zur häuslichen Pflege und stärkere Präferenzen für Heime oder Wohngruppen im Vergleich zu der eigenen Wunschpräferenz [5]. Diagnostizierte Demenz stellt einen deutlichen Risikofaktor für eine Aufnahme in ein Pflegeheim dar, auch weil pflegende Angehörige von Menschen mit Demenz einer besonders hohen Belastung ausgesetzt sind [12].

Die erwarteten Veränderungen durch einen Heimeinzug waren negativ für die Mehrheit älterer Befragter in einer früheren Studie [3]. Verschlechterungen wurden hinsichtlich der Selbstständigkeit, Kontakten zu Personen außerhalb des Pflegeheims, der Zimmereinrichtung und der finanziellen Situation erwartet. Je weniger klare Vorstellungen eine ältere Person vom Pflegeheim hatte, desto größer waren die Befürchtungen. Nach einer weiteren früheren Befragung haben sich 80 % der Befragten selten oder nie mit Pflegeheimen beschäftigt [13].

Viele Ältere haben keine angemessenen Informationen über das Leben im Heim [20]. Dadurch können Ängste und negative Stereotype, die in der Gesellschaft vorherrschen, nicht widerlegt werden. Medienberichte, in denen von Missbrauch, unfähigem Personal oder Profitmaximierung gesprochen wird, formen die Vorstellungen älterer Menschen [28, 29] und können eine Abwehrhaltung gegenüber Pflegeheimen bewirken [1]. Zudem geht aus dem DAK-Pflegereport (2018) hervor, dass die Mehrheit der Bevölkerung die Pflege im Heim als zu teuer ansieht und als wenig angenehm für die Gepflegten [5].

Umfassendere Informationen und direkte Erfahrungen können dagegen positivere Einstellungen befördern und Ängste reduzieren. Ältere sahen das Pflegeheim eher als eine annehmbare Option an, wenn die eigenen Eltern bereits in einem Pflegeheim gelebt haben [18]. Auch andere Studien machen deutlich, dass Erfahrung einen positiven Einfluss auf die Einstellung zum Pflegeheim haben kann [21]. Ältere, die bereits häufiger Kontakt zu einem Pflegeheim hatten, präferierten Pflegeheime mit größerer Wahrscheinlichkeit (OR = 1,93).

Neben der Informiertheit und den Erfahrungen stellen zudem die Autonomie, als Ausdruck von Selbstbestimmung und Wahlfreiheit, und die wahrgenommene Kontrolle entscheidende Determinanten für das Bewältigen der Entwicklungsaufgabe des Heimeinzugs dar [8]. Das Erleben von Kontrolle über den Umzug erhöht die Wahrscheinlichkeit für Wohlbefinden nach einem Heimeinzug deutlich (OR = 3,64) [25]. Auch qualitative Ergebnisse bestätigen die Bedeutung von Kontrollerleben für die Anpassung an einen Heimeinzug [2]. Diese Befunde sind gerade im Hinblick darauf wichtig, dass in der Mehrheit der Fälle die Betroffenen nicht am Entscheidungsprozess beteiligt sind [4, 11]. Bei einer schriftlichen Befragung von Angehörigen und Hausärzten wurde angegeben, dass in nur ca. 30 % der Fälle die betroffene Person an der Entscheidung beteiligt war [4]. Auch wenn zu beachten gilt, dass die betroffenen Personen eine leichte bis mittelschwere Demenz aufwiesen.

In der hier berichteten Studie wurden Patienten in geriatrischer Rehabilitation zu ihrer Einstellung zu Pflegeheimen befragt, und es wurde Zusammenhängen der Einstellung mit dem Informationsstand, Erfahrungen und dem Kontrollerleben der Befragten nachgegangen. Erweisen sich Informationsstand und Erfahrungen wie erwartet als bedeutsam, bieten sich Ansatzpunkte für Interventionen, um psychische Belastung zu senken, wenn der Umzug in ein Pflegeheim erwogen werden muss. Zeigen sich zudem positivere Einstellungen bei höherem Kontrollerleben, verweist das auf die Möglichkeit, durch stärkere Beteiligung der Betroffenen eine weniger belastende und offenere Auseinandersetzung mit der Option eines Heimeinzugs zu fördern.

## Methode

### Stichprobe

Insgesamt umfasst die Stichprobe 150 deutschsprachige Probanden (115 weiblich) im Alter zwischen 62 und 96 Jahren (*M* = 82,21 ± 6,48 Jahre). Die Probanden befanden sich zum Zeitpunkt der Erhebung in geriatrischer Rehabilitation. Auch wenn die Mehrheit, 85,8 % der Patienten (Geriatriezentrum Chemnitz, 2017), wieder nach Hause zurückkehrt, werden sich viele früher oder später mit Gedanken an eine passende Wohnform im höheren Alter auseinandersetzen müssen – für sich selbst oder auch für eine nahestehende Person. Die Zeit in der Rehabilitation bietet zudem einen ersten Einblick in eine stationäre Wohnform und stellt einen guten Rahmen dar, das Thema Pflegeheim aufzugreifen.

### Befragungsinstrument

**Figure 1:**
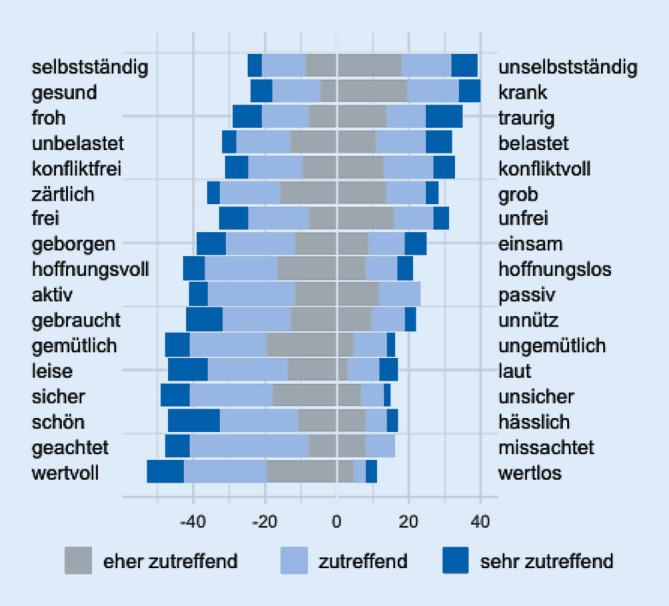

Die Geriatriepatienten wurden in Einzelinterviews mit standardisierten und offenen Items zu ihrer Einstellung gegenüber Pflegeheimen, ihren Vorstellungen, ihrem Informationsstand, ihren Erfahrungen und ihrem Kontrollerleben befragt. Im Interview wurde der im Alltagssprachgebrauch gängige Begriff „Altenheim“ verwendet. Die Befragten äußerten keine Verständnisschwierigkeiten, und auch ihre freien Antworten ließen erkennen, dass sie darunter, wie intendiert, stationäre Pflegeeinrichtungen verstanden. Nach den ersten 64 Interviews wurde die Befragung etwas modifiziert (Tab. 1). Beispielsweise wurden die Antwortalternativen pro Adjektivpaar im Polaritätsprofil (Abb. 1) aufgrund häufiger Schwierigkeiten mit dem 6‑stufigen Antwortformat zur Wahl zwischen 2 Adjektiven reduziert. Dazu wurden die einzelnen Adjektive auf Kärtchen geschrieben, das zusammengehörige Wortpaar wurde hochgehalten, und die Personen wählten, welches Wort sie eher mit einem Pflegeheim verbinden würden. In der ersten Befragungsversion wurde nicht näher auf Angst eingegangen, eine ängstliche Einstellung wurde aber öfter deutlich. Um die Angst in ihrer Stärke und mögliche Ursachen genauer zu erfassen, wurden in der zweiten Version 4 Items zu Ängstlichkeit ergänzt. Angst wurde in dieser Befragung nach dem State-Trait-Angstinventar (STAI) [9] nicht als überdauernde Persönlichkeitseigenschaft (Trait) erfasst, sondern vielmehr ging es um die Erfassung der Angst als aktuellen Zustand (State). Die Angst bezieht sich hier auf eine bestimmte Situation, den möglichen Umzug in ein Pflegeheim. Weiterhin hatte das Altersselbstbild nach den ersten Ergebnissen keinen Mehrwert für die Studie und wurde in der zweiten Version nicht mehr erfragt. Mit dem modifizierten Instrument wurden weitere 86 Probanden befragt. In Tab. 1 sind die übereinstimmenden Elemente der beiden Befragungsversionen zu erkennen.

**Table 1:** 

Erste Fragebogenversion	Zweite Fragebogenversion
*1. Demografische Angaben*	*1. Demografische Angaben*
*2. Allgemeine Einstellung zum Pflegeheim*	*2. Allgemeine Einstellung zum Pflegeheim*
Polaritätsprofil mit Antwortkategorien^a^	**Polaritätsprofil ohne Antwortkategorien**^**b**^
Haltung zum Umzug	Haltung zum Umzug
Wahrscheinlichkeit des Heimeinzugs	**Vier Items zur Ängstlichkeit**
	Offene Frage zu möglichen Ansätzen und Angeboten
	Wahrscheinlichkeit des Heimeinzugs
*3. Vorstellungen über Pflegeheime*	*3. Vorstellungen über Pflegeheime*
11 stereotype Aussagen	11 stereotype Aussagen
Offene Frage zu Stereotypen	Offene Frage zu Stereotypen
*4. Informationsstand und Erfahrung bezüglich Pflegeheimen*	*4. Informationsstand und Erfahrung bezüglich Pflegeheimen*
Drei Items zum Besuch	**Zwei Items zum Besuch**
Zwei Items zu Kostenvorstellungen	**Drei Items zum Informationsstand**
Zwei Items zum Informationsstand	
Offene Frage zu Beschäftigungen von Heimbewohnern	
*5. Kontrollerleben*	*5. Kontrollerleben*
Vier Items	Vier Items
*6. Altersbild*	*6. Kosten des Pflegeheims*
Acht Items	**Vier Items zu Kostenvorstellungen** (im Fragebogen vor den Items zu Kontrollerleben)
*7. Vorschläge*	
Offene Frage zu möglichen Ansätzen und Angeboten	

Änderungen sind fett ausgezeichnet

^a^Übernommen von Thiele et al. [27]

^b^Mit Antwortkärtchen

## Ergebnisse

Die Ergebnisse des Polaritätsprofils zur Einstellung zum Pflegeheim („Altenheim“) der ersten Befragungsversion mit 6 abgestuften Antwortalternativen (sehr zutreffend, zutreffend, eher zutreffend für das eine und das andere Adjektiv eines Gegensatzpaares) zeigt Abb. 1. Der Großteil der Probanden wählte eher positive als negative Adjektive. Bei 5 von 17 Wortpaaren wurde das negative Adjektiv (unselbstständig, krank, traurig, belastet, konfliktvoll) als eher zutreffend eingeschätzt.

In der zweiten Version des Fragebogens wurde auf die gestuften Antwortkategorien verzichtet und die Probanden wählten nur zwischen den gegenübergestellten Adjektiven. Dies ergab ein ähnliches Bild. Bei 5 von 17 Begriffspaaren haben sich die Probanden häufiger für das negative Adjektiv entschieden, mit Anteilen zwischen 53,5 % und 61,6 %.

Im Anschluss an die Einschätzungen von Aussagen waren die Probanden aufgefordert, eigene Begriffe und Aussagen aufzuschreiben, die sie mit einem Pflegeheim („Altenheim“) verbinden. Insgesamt wurden 229 freie Antworten gesammelt. Die Antworten wurden gemäß dem Ablaufmodell zusammenfassender Inhaltsanalyse in negativ, positiv und neutral kategorisiert [16]. Im Unterschied zu den Ergebnissen des Polaritätsprofils überwogen in den freien Antworten deutlich die negativen Begrifflichkeiten. Von den 229 freien Antworten lassen sich 153 Aussagen (66,8 %) unter eher negativ gepolten Begriffen zusammenfassen. Die meisten Aussagen lassen sich den Kategorien Abwehrhaltung (z. B. „Ich kann den Geruch schon nicht ertragen.“) oder Unselbstständigkeit und Abhängigkeit von anderen (z. B. „Da kann ich gar nichts mehr.“) zuordnen. Weitere 50 Aussagen (21,8 %) waren positiv, und 26 Aussagen (11,3 %) wurden als neutral gewertet.

Die Frage nach der Haltung zum Umzug in ein Pflegeheim („Altenheim“) wurde allen Probanden gestellt und ergab, dass knapp über die Hälfte der 150 Probanden eher ängstlich eingestellt sind (56 %), die übrigen sehen einen solchen Umzug als positive Herausforderung. Die Gründe für eine ängstliche Einstellung lassen sich den Kategorien schlechte Medienberichte und Erfahrungen (29,4 %) sowie der Aufgabe des gewohnten Umfelds (29,4 %) zuordnen. Über beide Haltungen zum Umzug hinweg glauben 73,3 %, die Kontrolle zu haben („Wenn Sie demnächst in ein Altenheim umziehen würden, glauben Sie, dass Sie die Kontrolle über die Entscheidung für oder gegen einen Umzug in ein Altenheim hätten?“). Fragt man nach den Verantwortlichen, dann geben 41,3 % an, dass sie glauben, Verwandte wären hauptsächlich für ihren Umzug in ein Pflegeheim verantwortlich, wohingegen 38,7 % sich selbst als verantwortlich ansehen und 20 % beziehen die Verantwortlichkeit für den eigenen Heimeinzug auf Ärzte.

Über die Hälfte der Probanden (70 %) haben angegeben, dass sie sich nicht ausreichend über Pflegeheime informiert fühlen („Wenn Sie nächste Woche in ein Altenheim umziehen sollten, fühlten Sie sich dann ausreichend über den Umzug und den Alltag im Altenheim informiert?“). Unter den 89 Probanden (59,3 %), die ein Pflegeheim besucht haben, herrschte für 64 % ein positives Bild vor.

Zusammenhängen der dichotom bestimmten Haltung zum Umzug mit Kontrollerleben, Informiertheit und Besuchserfahrungen sind wir mit logistischen Regressionen nachgegangen. Eine positive Haltung zum möglichen Umzug in ein Pflegeheim war häufiger unter männlichen Befragten (58 % vs. 36 % unter Frauen) und war wahrscheinlicher beim Vorliegen von subjektivem Kontrollerleben über den Umzug, subjektiver Informiertheit und einem positiven Bild aus Besuchserfahrungen (Tab. 2). Bloße Besuchserfahrungen erhöhten die Wahrscheinlichkeit einer positiven Haltung nicht (*b* = 0,04 für Besuchserfahrungen als alleiniger Prädiktor, *z* = 0,12, *p* = 0,91). Alter hatte ebenfalls keine Erklärungskraft (*b* < 0,001 für Alter als alleiniger Prädiktor, *z* < 0,01, *p* = 0,996).

**Table 2:** 

	*b*	*SE*	*z*	*p*	OR [95 %-KI]
Konstante	−1,82	0,45	–	–	–
Männliches Geschlecht	0,81	0,43	1,89	0,06	2,26 [0,98; 5,32]
Kontrollerleben	0,88	0,44	2,03	0,04	2,42 [1,06; 5,90]
Informiertheit	0,72	0,40	1,83	0,07	2,06 [0,95; 4,52]
Pos. Besuchserfahrung	0,93	0,37	2,52	0,01	2,54 [1,24; 5,31]

*R*^*2*^ = 0,11 (Hosmer-Lemeshow), 0,14 (Cox-Snell), 0,18 (Nagelkerke). Modell χ^2^(4) = 20,87, *p* < 0,001

Für einen Großteil der Probanden spielen die Kosten bei der Auswahl des Pflegeheims eine Rolle (79,3 %), und sie würden das bevorzugte Pflegeheim anhand der Kosten auswählen (76,7 %). Wenn es allerdings um Verbesserungen der Pflegeheime geht, dann werden zum Großteil Begrifflichkeiten genannt, die sich dem Personal (z. B. „mehr Personal“) oder den wohnlichen Gegebenheiten und Ausstattungen (z. B. „Einzelzimmer“) zuordnen lassen. Lediglich 4 Anmerkungen wurden in Bezug auf weniger Kosten bei Pflegeheimen gemacht.

Viele freie Bemerkungen der Probanden und der Angesprochenen, die nicht teilnahmen (30 %), ließen eine starke Abwehrhaltung gegenüber Pflegeheimen erkennen. So war die Begründung für die Nichtteilnahme einiger Älterer, dass das Pflegeheim nie eine Option wäre und man sich demnach keine Gedanken darüber machen müsse. Manche reagierten zudem verärgert, wenn nach Pflegeheimen gefragt wurde, oder zeigten kein Verständnis dafür, dass es um die generelle Haltung zu Pflegeheimen geht, unabhängig davon, ob das für sie in der Zukunft eine Möglichkeit darstellt.

## Diskussion

Patienten in geriatrischer Rehabilitation äußerten im freien Antwortformat überwiegend negative Einstellungen gegenüber Pflegeheimen und gaben mehrheitlich an, sich nicht ausreichend informiert zu fühlen und eine ängstliche Einstellung zu haben. Im Gegensatz zu mehrheitlich negativen Einstellungen im freien Antwortformat, hat sich im semantischen Polaritätsprofil ein überwiegend positives Bild ergeben.

Eine mögliche Erklärung für das positive Stimmungsbild im Polaritätsprofil ist, dass Wünsche und Sollzustände in die Bewertung eingeflossen sind. Des Weiteren äußerten einige Probanden bei der Bearbeitung des Polaritätsprofils, dass das Pflegeheim sicher eine gute Option für andere sei, aber nicht für sie selbst. Zu Bedenken ist weiterhin, dass die Probanden, welche eine starke Abwehrhaltung gegenüber einem Pflegeheim haben, nicht befragt werden konnten. Wäre es möglich, diesen Anteil an Personen einzubeziehen, so würde das Polaritätsprofil negativer ausfallen.

Im Einklang mit den überwiegend negativen, frei formulierten Begriffen und Aussagen steht das Ergebnis, dass der Großteil der Befragten mit 56 % eine eher ängstliche Haltung gegenüber dem Pflegeheim hat. Gerade die Aufgabe des gewohnten Umfelds, der Verlust der Selbstständigkeit und schlechte Medienberichte wurden als Gründe für die Angst genannt und sind konsistent mit früheren Befunden [1]. Kontrollerleben hängt hingegen mit einer positiveren Einstellung zum Pflegeheim zusammen, und die Mehrheit (73,3 %) glaubt, die Kontrolle über die Entscheidung zu haben. Doch es ist fraglich, ob die Personen zum Zeitpunkt des Umzugs wirklich die Kontrolle haben. In den meisten Fällen sind die Betroffenen nicht am Entscheidungsprozess beteiligt [4]. Bei einer schriftlichen Befragung von Angehörigen und Hausärzten wurde angegeben, dass in nur ca. 30 % der Fälle die betroffene Person an der Entscheidung beteiligt war [4]. Es gilt zu beachten, dass die betroffenen Personen in jener Studie eine leichte bis mittelschwere Demenz aufwiesen. Von den Angehörigen waren 72 % Schwieger‑/Kinder und 23 % Ehepartner [4]. Der Zusammenhang zwischen positiven Erfahrungen und der Bewertung des Pflegeheims konnte in der vorliegenden Untersuchung belegt werden. Ähnlich wie in früheren Studien fühlten sich 70 % der Befragten nicht ausreichend über Pflegeheime informiert [13, 20]. Es konnte darüber hinaus gezeigt werden, dass Personen, die sich unzureichend informiert fühlen, ängstlicher gegenüber dem Pflegeheim sind. Es wäre denkbar gewesen, dass im Vergleich zu älteren Studien der Anteil der Informierten gestiegen ist. Über die Jahre sollte die Entwicklung der Informationsbereitstellung und Beratung zu Wohnformen im Alter einen positiven Einfluss auf die Informiertheit haben. Gleichzeitig ist es aber auch denkbar, dass sich hauptsächlich die Angehörigen zu besagten Angeboten informieren. In einer Studie suchten lediglich 3,1 % für sich selbst Informationen bezüglich Pflegeheimen [11].

Die befragten Männer gaben häufiger eine positive Haltung zum möglichen Umzug in ein Pflegeheim an. In früheren Studien finden sich keine Hinweise auf diesen Geschlechtsunterschied [21, 22]. Da Männer den kleineren Teil der Stichprobe bilden (35 vs. 115 Frauen), wäre es möglich, dass der Befund nicht reliabel ist. Eine tatsächlich positivere Haltung könnte z. B. auf geringere wahrgenommene soziale Unterstützung zurückgehen. In einer früheren Untersuchung nannten Männer insgesamt weniger Bezugspersonen und Unterstützungspotenzial [14].

Die Ergebnisse der Studie lassen sich aufgrund der ausgewählten Stichprobe nicht auf die Gesamtheit Älterer übertragen, da die Probanden teilweise für mehrere Wochen nicht zu Hause waren und teilweise von mehreren Operationen geschwächt und desorientiert in der Einrichtung ankamen. Der vermutlich unterdurchschnittliche subjektive Gesundheitszustand könnte die Einstellungen zu Pflegeheimen beeinflusst haben. Dies konnte am vorliegenden Datensatz nicht direkt überprüft werden. In früheren Studien hatten soziodemografische Variablen wie Alter, Geschlecht oder Einkommen und gesundheitliche Aspekte keinen Einfluss auf die Meinung über Pflegeheime [22]. Und auch die Umzugsneigung wurde von subjektiv empfundenen Einschränkungen nicht beeinflusst [19].

Trotz der nichtrepräsentativen Stichprobe kann diese Studie Aufschluss darüber geben, welche Aspekte im Vorfeld bei einem möglichen Heimeinzug bedacht werden sollten. Gerade bei Personengruppen mit kognitiven Einschränkungen wie Demenz ist die Wahrscheinlichkeit für einen Heimeinzug erhöht [12]. Lediglich 20–40 % der Personen mit Demenz werden bis zu ihrem Tod zu Hause gepflegt [4]. Nach einer Befragung von Heimleitern ist der häufigste Weg in das Pflegeheim mit 60,2 % über das Krankenhaus, gefolgt von 27,1 % über die Warteliste und mit 8,3 % über eine vorherige ambulante Betreuung [23]. Demnach können die Ergebnisse dieser Studie v. a. für Personen relevant sein, die nach einem Krankenhausaufenthalt in eine Pflegeeinrichtung umziehen. Hier ist allerdings zu beachten, dass nach dem Krankenhausaufenthalt auch die Kurzzeitpflege in Betracht kommen kann [23]. Von den Pflegebedürftigen in der Kurzzeitpflege waren 40,8 % vorher im Krankenhaus [23]. Zu betonen ist, dass eine Pflegeeinrichtung nur eine mögliche Option ist. Die Pflege im Haushalt zu stärken, ist ein wichtiges pflegepolitisches Ziel [6]. Unterstützungsleistungen wie Tages- oder Nachtpflege können hinzugezogen werden. Im Jahr 2017 bezog der Großteil Pflegebedürftiger Pflegegeld (54,5 %), ein Fünftel beanspruchte Sachleistungen (wie Pflegedienst) oder eine Kombination aus Geld- und Sachleistungen (21,1 %), und jeder vierte Pflegebedürftige lebte in einem Pflegeheim [7].

In Bezug auf die Wohnoption Pflegeheim wird mit den Ergebnissen der vorliegenden Studie sichtbar, dass viele negative freie Assoziationen zu Pflegeheimen existieren und Pflegeheimen oftmals mit einer ängstlichen, nichtinformierten Haltung begegnet wird. Um Pflegeheime noch transparenter darzustellen, gibt es seit 01.10.2019 ein neues Qualitäts- und Prüfsystem in der stationären Pflege (interne Qualitätsdaten) und seit 01.11.2019 eine neue Qualitätsprüfung durch den Medizinischen Dienst der Krankenversicherung (MDK) [15]. Damit wird die kritisierte Pflegenotendarstellung abgelöst, und die Interessierten erhalten umfassendere Informationen über Pflegeheime. Die ersten Prüfergebnisse sind bereits vorhanden, und es gilt abzuwarten, wie Interessierte die neue Darstellung bewerten.

Wenn immer möglich sollten die Betroffenen einbezogen, über neueste Entwicklungen informiert und am Entscheidungsprozess beteiligt werden. Die Frage nach individuell passender pflegerischer Versorgung und Qualität nimmt unter der wachsenden Anzahl Pflegebedürftiger zu [26]. Der Entscheidungsprozess ist vielschichtig und umfasst Informationen zu Versorgungsmöglichkeiten und finanziellen Leistungen sowie das Klären von Anforderungen an eine Wohnalternative im Alter. Die Information und Beteiligung der Betroffenen sollte proaktiv geschehen, denn mit der Vielzahl an internetbasierten Informations- und Beratungsangeboten können hauptsächlich Angehörige erreicht werden [26].

## Fazit für die Praxis

- Die Ergebnisse der vorliegenden Studie machen deutlich, dass viele negative freie Assoziationen zu Pflegeheimen existieren und ihnen oftmals mit einer ängstlichen Haltung begegnet wird. Zudem besitzt die Mehrheit der Probanden keine adäquaten Informationen über Pflegeheime.
- Die Entscheidung über Pflegearrangements sollte bestmöglich informiert und, wenn immer möglich, unter Beteiligung der Betroffenen getroffen werden. Proaktive Information und Beteiligung sind nötig, um ungerechtfertigte Ängste zu nehmen, Abwehrhaltungen entgegenzuwirken und im Fall des Umzugs in eine Pflegeeinrichtung die Integration in die neuen Wohnverhältnisse zu erleichtern.
